# Characteristics and predictors for gastrointestinal hemorrhage among adult patients with dengue virus infection: Emphasizing the impact of existing comorbid disease(s)

**DOI:** 10.1371/journal.pone.0192919

**Published:** 2018-02-20

**Authors:** Wen-Chi Huang, Ing-Kit Lee, Yi-Chun Chen, Ching-Yen Tsai, Jien-Wei Liu

**Affiliations:** 1 Division of Infectious Diseases, Department of Internal Medicine, Kaohsiung Chang Gung Memorial Hospital, Kaohsiung, Taiwan; 2 Chang Gung University College of Medicine, Tao-Yuan, Taiwan; Centers for Disease Control and Prevention, UNITED STATES

## Abstract

**Background:**

Gastrointestinal (GI) bleeding is a leading cause of death in dengue. This study aims to identify predictors for GI bleeding in adult dengue patients, emphasizing the impact of existing comorbid disease(s).

**Methods:**

Of 1300 adults with dengue virus infection, 175 (mean age, 56.5±13.7 years) patients with GI bleeding and 1,125 (mean age, 49.2±15.6 years) without GI bleeding (controls) were retrospectively analyzed.

**Results:**

Among 175 patients with GI bleeding, dengue hemorrhagic fever was found in 119 (68%) patients; the median duration from onset dengue illness to GI bleeding was 5 days. Gastric ulcer, erythematous gastritis, duodenal ulcer, erosive gastritis, and hemorrhagic gastritis were found in 52.3%, 33.3%, 28.6%, 28.6%, and 14.3% of 42 patients with GI bleeding who had undergone endoscopic examination, respectively. Overall, nine of the 175 patients with GI bleeding died, giving an in-hospital mortality rate of 5.1%. Multivariate analysis showed age ≥60 years (cases vs. controls: 48% vs. 28.3%) (odds ratio [OR]: 1.663, 95% confidence interval [CI]: 1.128–2.453), end stage renal disease with additional comorbidities (cases vs. controls: 1.7% vs. 0.2%) (OR: 9.405, 95% CI: 1.4–63.198), previous stroke with additional comorbidities (cases vs. controls: 7.4% vs. 0.6%) (OR: 9.772, 95% CI: 3.302–28.918), gum bleeding (cases vs. controls: 27.4% vs. 11.5%) (OR: 1.732, 95% CI: 1.1–2.727), petechiae (cases vs. controls: 56.6% vs. 29.1%) (OR: 2.109, 95% CI: 1.411–3.153), and platelet count <50×10^9^ cells/L (cases vs. controls: 53.1% vs. 25.8%) (OR: 3.419, 95% CI: 2.103–5.558) were independent predictors of GI bleeding in patients with dengue virus infection.

**Conclusions:**

Our study is the first to disclose that end stage renal disease and previous stroke, with additional comorbidities, were strongly significant associated with the risk of GI bleeding in patients with dengue virus infection. Identification of these risk factors can be incorporated into the patient assessment and management protocol of dengue virus infection to reduce its mortality.

## Introduction

Dengue is the most common mosquito-borne arboviral disease in the world [1]. The World Health Organization (WHO) estimates that 50–100 million people are infected annually and over 2.5 billion people living in tropical and subtropical regions worldwide are at risk [2]. The clinical manifestations of dengue virus (DENV) infection vary greatly, ranging from asymptomatic, self-limiting febrile illness, dengue fever (DF) and dengue hemorrhagic fever (DHF) to dengue shock syndrome (DSS), the most severe form of DENV infection [1,2]. Of note, the number of dengue cases is increasing globally, both among travelers as well as those residing in endemic regions [1,2]. One of the most vital challenges for physicians caring for dengue patients is the early identification of those in whom the disease will evolve to its severe form. Remarkably, a variety of dengue-related complications have been reported in dengue-affected patients, with bleeding as one of the major ones, which contributes to its morbidity and mortality [3–7]. In 2009, the WHO released revised dengue guidelines that proposed mucosal bleeding as one of warning signs of severe dengue, and severe bleeding as one category of severe dengue [8]. The most common bleeding manifestations in dengue are epistaxis, gum bleeding, and cutaneous hemorrhages [1]; however, gastrointestinal (GI) bleeding was reported to be an indicator of poor prognosis in dengue-affected patients [5–7]. Sam et al [7] noted that 56% of fatal cases experienced GI bleeding, and other reports [3] have shown that 45.5% of fatal patients had GI bleeding. Yet, little information is available about the risk factors for GI bleeding in adult patients with DENV infection [9,10]. Early recognition of risk factors for GI bleeding and prompt initiation of appropriate treatment is important in the treatment of dengue-affected patients, which in turn can potentially reduce morbidity and mortality. In this report, we aimed to describe the clinical and laboratory presentations of patients with GI bleeding and identification of clinical predictors associated with GI bleeding in adult dengue-affected patients, emphasizing the impact of existing comorbid disease(s). Our findings should be helpful to physicians regarding the decision of hospital admission for a patient with DENV infection and providing timely management, particularly in resource-poor settings.

## Methods and materials

### Ethics statement

This study was approved by the Institutional Review Board of Kaohsiung Chang Gung Memorial Hospital (KSCGMH) (Document No. 201700059B0), Taiwan. Informed consent was not required as the data were analyzed anonymously.

### Patient and dengue diagnostics

All adult patients (≥18 years) with laboratory-confirmed DENV infection from 2002 to 2013 at KSCGMH, a 2,500-bed facility serving as a primary care and tertiary referral center in Taiwan, were identified using our hospital database and retrospectively reviewed. Children (<18 years old) and adult patients with missing data were excluded from analysis. All patients with DENV infection included in this study were confirmed by at least one of the following criteria: (i) positive DENV−specific real-time reverse transcription polymerase chain reaction (RT-PCR; QuantiTect SYBR Green RT-PCR kit; Qiagen, Hilden, Germany), (ii) a fourfold increase in DENV−specific immunoglobulin G (IgG) antibody in the convalescent serum compared to in the acute-phase serum, and/or (iii) detection of DENV−specific nonstructural glycoprotein-1 antigen (Bio-Rad Laboratories, Marnes-la-Coquette, France) in the acute-phase serum [11,12]. The Center for Disease Control, Taiwan, performed all diagnostic laboratory tests.

### Dengue illness severity

Dengue-affected patients in this study were classified as DF and DHF based on 1997 WHO definitions [13]. The diagnosis of DHF was established based on the presence of fever, hemorrhage, thrombocytopenia (platelet count <100 × 10^9^ cells/L), and clinical evidence of plasma leakage (presence of hemoconcentration, pleural effusion, ascites, and/or hypoalbuminemia). DHF grades 1 and 2 were defined as a positive tourniquet test result being the only hemorrhagic manifestation and the occurrence of spontaneous bleeding such as mucosal bleeding, respectively. DHF grades 3 and 4 were grouped as DSS, defined as cases of DHF with circulatory failure [13]. The 2009 WHO dengue classification was not utilized in this series because 65.1% of patients with DENV infection were enrolled during 2002–2008, prior to the introduction of the revised 2009 classification [8].

### Definitions

GI bleeding was defined as hematemesis and/or passage of tarry or bloody stool. Massive GI bleeding was defined as GI bleeding coupled with hemodynamic instability (systolic blood pressure <90 mm Hg) and/or a rapid drop in hemoglobin (hemoglobin level <10 g/dL) within 48 h [14]. Leukocytosis was defined as a peripheral white blood cell count (WBC) >10 × 10^9^ cells/L, and leukopenia as a peripheral WBC <3.0 × 10^9^ cell/L (reference value, 3.0–10 × 10^9^ cells/L) [15]. The severity of thrombocytopenia was stratified as (i) platelet count >100 ×10^9^ cells/L, (ii) platelet count between 50 and 99 × 10^9^ cells/L and (iii) platelet count <50 × 10^9^ cells/L. A comorbidity was defined as an underlying chronic disease that had been diagnosed in a patient prior to the development of the dengue illness such as type 2 diabetes mellitus, essential hypertension, non-dialysis chronic kidney disease (estimated glomerular filtration rate <60 mL/min/1.73 m^2^ for 3 months), end stage renal disease requiring hemodialysis, previous stroke, and ischemic heart disease. In-hospital mortality was defined as death occurring during the hospital stay for dengue illness.

### Data collection

For each included patient, information on the patient’s demographic, clinical, laboratory and outcome data were recorded in a standardized form. To disclose the predictive factors for GI bleeding, the day that the patients fulfilled the criteria for GI bleeding was determined, and the clinical signs/symptoms as well as the results of laboratory tests and radiography/ultrasound examinations before onset of GI bleeding was retrieved for analyses. For dengue-affected patients without GI bleeding, data at admission were used in the study. The hospital electronic medical records were used to extract the data and were supplemented by a secondary manual search.

### Statistical analysis

Patients were categorized as those either with GI bleeding or without GI bleeding. We compared the demographic, clinical, and laboratory data of the patients with and without GI bleeding. Mann-Whitney U tests and Fisher’s exact tests were used to determine statistical significance for continuous and categorical variables, respectively. A two-tailed P < 0.05 was considered statistically significant. A variable with P < 0.05 from univariate analysis were put into the full multivariate model. A multivariate logistic regression analysis using forwards selection was performed to identify variables independently associated with GI bleeding. SPSS statistical software (version 17.0; SPSS Inc., Chicago, IL) was used for all data analyses.

## Results

### Patient characteristics

**Fig 1:**
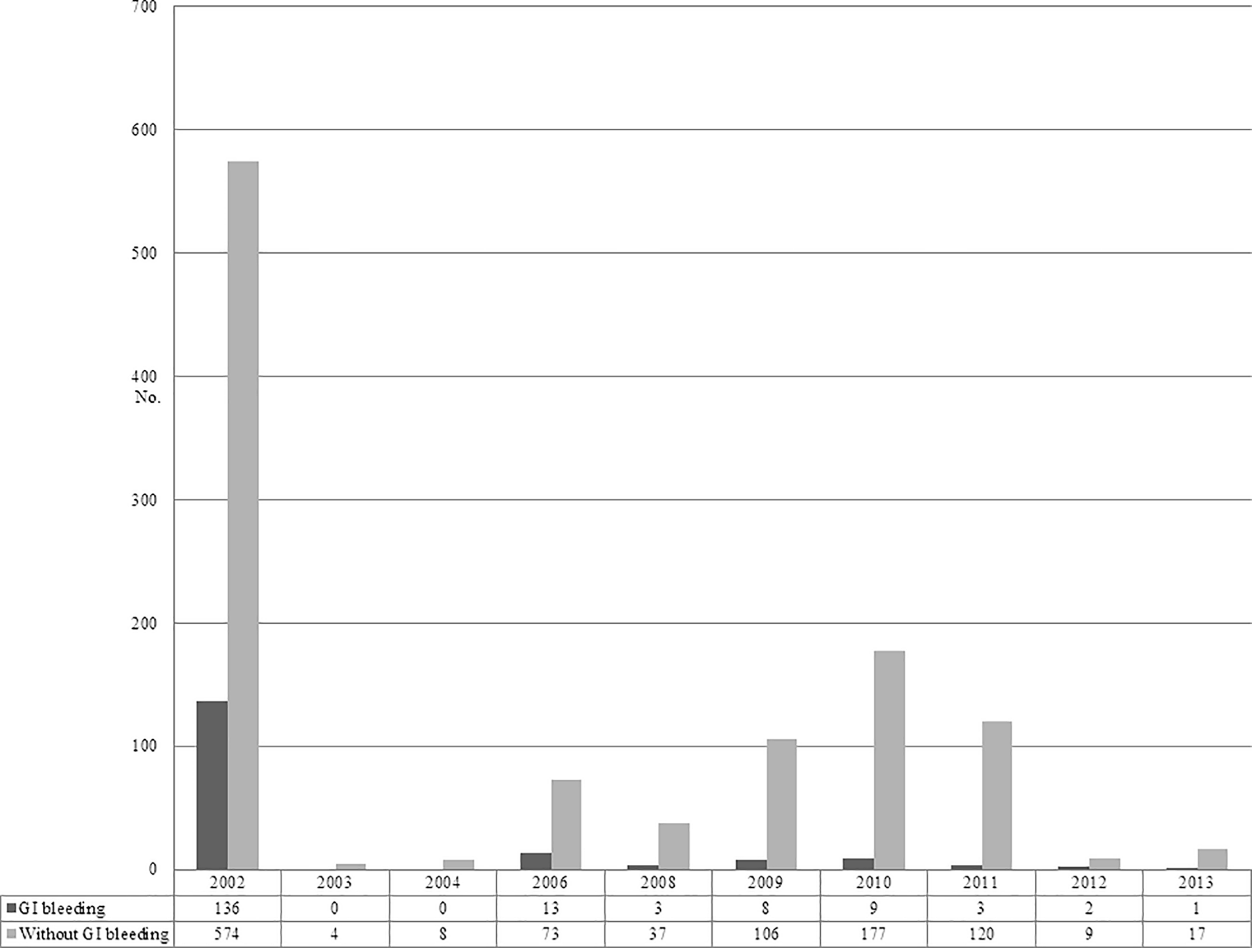
Number of dengue cases with and without gastrointestinal bleeding, 2002–2013.

During the study period (2002–2013), a large outbreak caused by DENV-2 occurred in 2002 (approximately 5300 cases identified) in southern Taiwan. The rest were sporadic dengue clusters and absence of large-scale dengue epidemics (Fig 1). Thus, most clinicians in Taiwan are not experienced in treating dengue patients, and the management of dengue was mainly based on their personal practice with treatment of febrile illness.

Ten adult dengue patients without GI bleeding were excluded owing to lacking of information regarding the demographic and historical data. As a result, 175 (13.5%) (86 men and 89 women) patients with GI bleeding and 1,125 (86.5%) (520 men and 605 women) without GI bleeding were included in our series. The information with respect to the drugs a patient uses prior to hospital admission was not obtained from the medical record. None of the patients taking aspirin or non-steroidal anti-inflammatory drugs during the hospital stay for dengue illness. A total of 937 (72%) patients had available RT-PCR data; among them, DENV-2 was detected in 823 (87.8%) patients, DENV-3 in 99 (10.6%), DENV-1 in 14 (1.5%), and DENV-4 in one (0.1%).

Among the 1125 patients without GI bleeding (mean age, 49.2 ± 15.6; 318 [28.3%] patients aged ≥60 years), 268 (23.8%) were DHF, and the three most common symptoms other than fever were bone pain (45.5%), headache (42.9%) and myalgia (35.9%). Of these patients, 2 (0.2%) patients received packed red blood cells transfusions owing to massive bleeding from the gums and nose. The in-hospital mortality rate was 0.3%, comprised of 3 patients.

Of 175 dengue cases with GI bleeding, 136 (77.7%) cases were enrolled in 2002 (Fig 1). Among 175 patients with GI bleeding (mean age, 56.5 ± 13.7; 84 [48%] patients aged ≥60 years), DHF was found in 119 (68%) patients; the three most frequently noted presentations other than fever before onset of GI bleeding were petechiae (56.5%), bone pain (51.4%), and headache (75%), and the median duration from onset dengue illness to GI bleeding was 5 (2–7) days. With regard to the laboratory data before onset of GI bleeding in the 175 patients, the mean platelet count was 62.3 ± 66.3 × 10^9^ cells/L. Of the 167 patients who had their hematocrit assayed before onset of GI bleeding, the mean hematocrit level was 37.7 ± 6.7%. Of note, pleural effusion was detected in 52 (38.5%) of 135 patients with an available chest radiography and/or ultrasonography result, and ascites in 38 (33.9%) of 112 patients with an available ultrasonography result. Forty-two patients received endoscopic examination while they experienced GI bleeding. Among the 42 patients (an individual might have more than one endoscopic finding), gastric ulcer was found in 22 patients (52.3%), erythematous gastritis in 14 (33.3%), duodenal ulcer in 12 (28.6%), erosive gastritis in 12 (28.6%), hemorrhagic gastritis in six (14.3%), duodenal erosion in four (9.5%), and esophageal ulcer in two (4.8%). History of peptic ulcer disease was reported in 6 (14.3%) of 42 patients received endoscopic examination. Massive GI bleeding developed in 24 (13.7%) (mean age, 63.7 ± 9.95 years; 18 [75%] patients aged ≥60 years) patients. Among the 24 patients with massive GI bleeding, all had been diagnosed with DHF, and seven (29.2%) patients died (S1 Table). Of 175 patients with GI bleeding, transfusion therapy with platelet concentrate in 128 (73.1%) patients, packed red blood cells in 38 (21.7%). Overall, nine of the 175 patients with GI bleeding died, including 6 patients died within one week after the onset of dengue illness, 2 died within 2 weeks, and one died within 4 weeks, giving an in-hospital mortality rate of 5.1%. The characteristic of the patients with and without GI bleeding is summarized in Tables 1 and 2.

**Table 1:** Characteristics and outcomes of dengue patients with and without GI bleeding.

Variable	Total patients (N = 1300)	GI bleeding (n = 175)	Without GI bleeding (n = 1125)	P^a^
Mean age ±SD, years	50.1 ± 15.5	56.5±13.7	49.2±15.6	<0.001
Age group, n (%)				
18–29 years	175 (13.5)	14 (8)	171 (15.2)	1
30–39 years	182 (14)	10 (5.7)	172 (15.3)	0.124
40–49 years	203 (15.6)	25 (14.3)	178 (15.8)	0.425
50–59 years	328 (25.2)	42 (24)	286 (25.4)	0.071
≥60 years	402 (30.9)	84 (48)	318 (28.3)	<0.001
Female, n (%)	694 (53.4)	89 (50.85)	605 (53.8)	0.471
Comorbid conditions, n (%)				
Type 2 diabetes mellitus only	80 (6.2)	12 (6.8)	68 (6)	>0.99
Hypertension only	159 (12.2)	18 (10.3)	141 (12.5)	0.458
Type 2 diabetes mellitus with hypertension only	99 (7.6)	13 (7.4)	86 (7.6)	>0.99
Non-dialysis chronic kidney disease only	6 (0.5)	2 (1.1)	4 (0.4)	>0.99
Non-dialysis chronic kidney disease with additional comorbidities	11 (0.8)	4^b^ (2.3)	7^c^(0.7)	>0.99
End stage renal disease (hemodialysis) with additional comorbidities	5 (0.3)	3^d^(1.7)	2^e^ (0.2)	0.020
Previous stroke with additional comorbidities	20 (1.5)	13^f^ (7.4)	7^g^ (0.6)	<0.001
Ischemic heart disease only	6 (0.5)	1 (0.6)	5 (0.4)	>0.99
Day from onset illness to hospital presentation, median (range)	3 (1–10)	4 (1–7)	3 (1–10)	0.028
Day from onset illness to GI bleeding, median (range)	―	5 (2–7)	―	―
DENV serotype, n/total n (%)				
DENV-1	14/937 (1.5)	2/150 (1.3)	12/787 (6.4)	>0.99
DENV-2	823/937 (87.8)	140/150 (93.3)	683/787 (86.8)	0.028
DENV-3	99/937 (10.6)	8/150 (5.3)	91/787 (11.5)	0.020
DENV-4	1/937 (0.1)	0/150 (0)	1/787 (0.1)	>0.99
Blood component transfusion, n (%)				
Platelet concentrate	465 (35.7)	128 (73.1)	337 (30)	<0.001
Packed red blood cells	40 (3.1)	38 (21.7)	2 (0.2)	<0.001
Dengue hemorrhagic fever, n (%)	387 (29.8)	119 (68)	268 (23.8)	<0.001
Grades 1 and 2	354 (27.2)	91 (52)	263 (23.4)	
Grades 3 and 4	33 (2.5)	28 (16)	5 (0.4)	
In-hospital mortality, n (%)	12 (0.9)	9 (5.1)	3 (0.3)	<0.001

DENV = dengue virus; GI = gastrointestinal bleeding; n/total n = number of patients/total number of patients with data available; SD = standard deviation; WHO = World Health Organization.

^a^ Comparison of the data between patients with and without GI bleeding.

^b^ Among the 4 patients with non-dialysis chronic kidney disease, hypertension, diabetes with hypertension, hypertension with ischemic heart disease, as well as diabetes with hypertension and ischemic heart disease each were found in one patient.

^c^ Among the 7 patients with non-dialysis chronic kidney disease, hypertension was found in 4 patients, and diabetes with hypertension, hypertension with ischemic heart disease as well as diabetes with hypertension and ischemic heart disease each were found in one.

^d^ Among the 3 patients with end stage renal disease, hypertension was found in one patient and diabetes with hypertension in 2.

^e^ Among the 2 patients with end stage renal disease, hypertension and diabetes with hypertension each were found in one patient.

^f^ Among the 13 patients with previous stroke, hypertension, diabetes, as well as diabetes with hypertension each were found in 4 patients, and diabetes with hypertension and ischemic heart disease in one.

^g^ Among the 7 patients with previous stroke, hypertension was found in 5 patients and diabetes with hypertension in 2.

**Table 2:** Symptoms/signs and laboratory characteristics of patients with and without GI bleeding.

Variable	GI bleeding^a^ (n = 175)	Without GI bleeding (n = 1125)	P
Symptom/sign,^b^ n (%)			
Fever	164 (93.7)	1072 (95.3)	0.370
Abdominal pain	68 (38.8)	267 (23.7)	<0.001
Orbital pain	24 (13.7)	126 (11.2)	0.333
Bone pain	90 (51.4)	512 (45.5)	0.144
Myalgia	52 (29.7)	404 (35.9)	0.110
Headache	75 (42.8)	479 (42.9)	0.945
Vomiting/nausea	63 (36)	342 (30.4)	0.137
Diarrhea	35 (20)	183 (16.3)	0.219
Petechiae	99 (56.5)	327 (29.1)	<0.001
Gum bleeding	48 (27.4)	130 (11.6)	<0.001
Epistaxis	5 (2.8)	13 (1.2)	0.073
Laboratory and images findings			
Leukopenia (WBC <3.0 × 10^9^ cells/L), n/total n (%)	33/171 (19.3)	371/1094 (33.9)	<0.001
Leukocytosis (WBC >10 × 10^9^ cells/L), n/total n (%)	9/171 (5.3)	14/1094 (1.3)	>0.99
Mean platelet count ±SD (× 10^9^ cells/L) (n)	62.3 ± 66.3 (n = 175)	98 ± 63.1 (n = 1100)	<0.001
Severity of thrombocytopenia			
Platelet count >100 × 10^9^ cells/L, n/total n (%)	39/175 (22.3)	533/1100 (48.5)	1
Platelet count 50–99 × 10^9^ cells/L, n/total n (%)	43/175 (24.6)	283/1100 (25.7)	0.002
Platelet count <50 × 10^9^ cells/L, n/total n (%)	93/175 (53.1)	284/1100 (25.8)	<0.001
Mean hematocrit ± SD (%) (n)	37.7 ± 6.7 (n = 167)	39.4 ± 5.8 (n = 1,088)	<0.001
Mean hemoglobin ± SD (g/dL) (n)	13 ± 2.4 (n = 156)	13.6 ± 1.8 (n = 1,017)	0.005
AST >1000 U/L, n/total n (%)	2/112 (1.8)	9/754 (1.2)	>0.99
ALT >1000 U/L, n/total n (%)	0/95 (0)	6/637 (0.9)	>0.99
Albumin <3 g/dL, n/total n (%)	15/40 (37.5)	20/199 (10)	>0.99
Pleural effusion, n/total n (%)	52/135 (38.5)	129/718 (18)	<0.001
Ascites, n/total n (%)	38/112 (33.9)	86/476 (18.1)	<0.001

ALT = serum alanine aminotransferase; AST = serum aspartate aminotransferase; GI = gastrointestinal bleeding; n/total n = number of patients/total number of patients with data available; WBC = white blood cell; SD = standard deviation.

^a^ Data before onset of GI bleeding were retrieved for analyses.

^b^ An individual patient might have more than one underlying disease/condition.

### Comparison of demographic, clinical and laboratory characteristics between dengue patients with and without GI bleeding

Comparisons between dengue patients with and without GI bleeding are shown in Tables 1 and 2. Compared to the patients without GI bleeding, patients with GI bleeding were of significantly higher age (P < 0.001), had end stage renal disease (hemodialysis) with additional comorbidities (see footnotes of Table 1 for details) (P = 0.020) and previous stroke with additional comorbidities (see footnotes of Table 1 for details) (P < 0.001), higher frequencies of DENV-2 infection (P = 0.028), packed red blood cells (P < 0.001) and platelet concentrate (P < 0.001) transfusion, as well as an increased incidences of DHF (P < 0.001), pleural effusion (P < 0.001), ascites (P < 0.001) and fatality (P < 0.001). Patients with GI bleeding had significantly higher frequencies of abdominal pain (P < 0.001), petechiae (P < 0.001) and gum bleeding (P < 0.001); lower platelet count (P < 0.001), hematocrit (P < 0.001) and hemoglobin (P = 0.005) levels in addition to lower incidence of leukopenia (P < 0.001) compared to patients without GI bleeding. Multivariate analysis revealed that older age (age ≥60 years) (odds ratio [OR]: 1.663, 95% confidence interval [CI]: 1.128–2.453; P = 0.010), end stage renal disease (hemodialysis) with additional comorbidities (OR: 9.405, 95% CI: 1.4–63.198; P = 0.021), previous stroke with additional comorbidities (OR: 9.772, 95% CI: 3.302–28.9182; P < 0.001), gum bleeding (OR: 1.732, 95% CI: 1.1–2.727; P = 0.018), petechiae (OR: 2.109, 95% CI: 1.411–3.153; P < 0.001), and platelet count <50 × 10^9^ cells/L (OR: 3.419, 95% CI: 2.103–5.558; P < 0.001) were independent predictors for GI bleeding in adult patients with DENV infection (Table 3).

**Table 3:** Multivariate analysis of independent risk factors associated with GI bleeding in patients with dengue.

	GI bleeding	Without GI bleeding	Odds ratio	95% confidence interval	P
Age ≥60 years					
No	91	807	1		
Yes	84	318	1.663	1.128–2.453	0.010
End stage renal disease (hemodialysis) with additional comorbidities^a^					
No	172	1123	1		
Yes	3	2	9.405	1.4–63.198	0.021
Previous stroke with additional comorbidities^a^					
No	162	1118	1		
Yes	13	7	9.772	3.302–28.918	<0.001
Gum bleeding					
No	127	995	1		
Yes	48	130	1.732	1.1–2.727	0.018
Petechiae					
No	76	798	1		
Yes	99	327	2.109	1.411–3.153	<0.001
Severity of thrombocytopenia					
Platelet count >100 ×10^9^ cells/L	39	533	1		
Platelet count 50–99 × 10^9^ cells/L	43	283	2.062	1.214–3.504	0.007
Platelet count <50 × 10^9^ cells/L	93	284	3.419	2.103‒5.558	<0.001

^a^See footnotes of Table 1 for details.

## Discussion

The incidence of GI bleeding in dengue patients was found to vary from 5% to 30% [16–18]. In our study, the prevalence of GI bleeding in adult patients with DENV infection during the study period was 13.5%. Notably, the fatality rate was 5.1% and 0.3% in adult dengue-affected patients with and without GI bleeding, respectively. Furthermore, once massive GI bleeding developed, the fatality rate can be as high as 29%. Our study highlights the urgent need for improving clinicians' awareness of this potentially fatal complication. Despite the time interval from dengue illness onset to presentation between the patients with and without GI bleeding differ significantly (median, 4 days vs. 3 days), the importance of continuous analyses of the relevant findings to assist clinicians early prediction of GI bleeding in dengue-affected populations cannot be overemphasized.

The spectrum of bleeding in dengue can range from minor mucosal bleeding such as epistaxis or gum bleeding to major bleeding. Major bleeding is almost always associated with profound shock, and is usually from the GI tract [5–7,19–21]. Previous studies reported that elderly patients with dengue had fewer hemorrhagic manifestations, particularly bleeding from the nose or gums, and petechiae [22]. However, even though fewer hemorrhagic manifestations are reported in the elderly, GI bleeding occurred more frequently among elderly patients than did other hemorrhagic manifestations [17]. Our findings here showed increased odds of GI bleeding in older adults with DENV infection. Notably, gastric ulcers were found endoscopically in 22 (52.3%) of 42 patients in our study. Although previous medication usage data were not available in this series, polypharmacy is common in the elderly with the increased use of aspirin or non-steroidal anti-inflammatory drugs for management of multiple comorbidities such as cardiovascular or rheumatologic diseases. As GI bleeding contributes to dengue-associated morbidity and mortality, our findings imply that caution needs to be exercised in older adults with DENV infection and timely appropriate management of GI bleeding is important to avoid otherwise preventable mortality and morbidity.

The 2009 WHO guidelines categorized disease severity into dengue without warning sign(s) (i.e., abdominal pain or tenderness, persistent vomiting, clinical fluid accumulation, mucosal bleed, lethargy or restlessness, liver enlargement >2 cm, and increase in hematocrit concurrent with rapid decrease in platelet count), dengue with warning sign(s), and severe dengue [8]. The presence of any warning sign is considered a key component for early recognition of potentially severe dengue [8]. Our analysis showed that gum bleeding and petechiae was independently predictors of GI bleeding in patients with DENV infection. Our report emphasized that these warning signs must be evaluated carefully and that patients exhibiting any of these warning signs need close monitoring, as these signs are potentially associated with GI bleeding that might evolve into life-threatening hemorrhage.

A relationship between end stage renal disease and GI bleeding in dengue-affected patients has not been described in the literature. In our study, we found that end stage renal disease with additional comorbidities was the strongest predictors of GI bleeding. End stage renal disease often coexists with other chronic conditions that may influence disease prognosis. The most prevalent comorbidities were hypertension and diabetes in those end stage renal disease patients, with or without GI bleeding (see footnotes of Table 1 for details). Remarkably, diabetes and hypertension, either alone or in combination, did not increase the risk of GI bleeding in our series. This finding highlights that end stage renal disease plays a key role in exacerbation of GI bleeding in dengue-affected patients. The exact reasons for the high rate of GI bleeding among dengue-affected patients with end stage renal disease are uncertain. However, it is proposed that pre-existing vascular disease and dialysis-specific factors such as heparin exposure during hemodialysis and platelet dysfunction resulting from uremia might contribute to the risk of GI bleeding [23–25]. Our study underlines that clinicians should be alert to possible GI bleeding when caring for a patient with end stage renal disease because this complication potentially leads to mortality if it is not recognized early enough and treated accordingly.

To our knowledge, this is the first study reporting that patients with previous stroke and coexisting other comorbidities were found to have 9-fold greater odds of development GI bleeding. Despite multiple comorbid conditions were noted in patients with previous stroke (see footnotes of Table 1 for details), we did not find a significant association between GI bleeding and the presence of diabetes, hypertension, or ischemic heart disease alone. Our result indicates that previous stroke appears to play a major causal role in the development of GI bleeding. The causes of GI bleeding in these patients is multifactorial, particularly medication-induced GI bleeding may be underappreciated in this setting. Of note, the strategy for reducing recurrent stroke using aspirin or oral anticoagulants [26,27] has been widely implemented in the health care system of the National Health Insurance in Taiwan. A systemic review reported that aspirin increased the odds of GI bleeding by threefold compared with placebo [28,29]. The combination of aspirin and clopidogrel increased the risk of GI bleeding compared with aspirin alone [28,29]. Further, warfarin is associated with an approximately threefold increased risk of GI bleeding as compared with the general population [28]. Even though the use of anticoagulants and aspirin were not evaluated in our series, it is not surprising that patients with cerebrovascular disease are given anticoagulants and aspirin for prevention of thromboembolic events. The addition of an aspirin or anticoagulants agent in the treatment of patients with previous stroke places them at greater risk of GI bleeding when suffering from dengue infection. Our finding highlights the importance of the careful assessment of the use of aspirin and anticoagulants in dengue-affected patients with a history of stroke and manages them accordingly.

A lower platelet count was significantly associated with GI bleeding in patients with DENV infection in our study. This finding was consistent with previous studies showing that patients with higher severities of thrombocytopenia have greater odds of bleeding [10,30]. Prophylactic platelet transfusions to prevent bleeding have not been shown to be effective in dengue infection [31]; however, blood transfusion is lifesaving and should be given as soon as severe bleeding is recognized [2].

Our findings are subject to several limitations. First, the retrospective nature of the study resulted in missing laboratory data regarding coagulation as well as information regarding the use of medications (such as aspirin and non-steroidal anti-inflammatory drugs) prior to hospital admission. Second, being a retrospective study, clinical outcomes of the GI patients may be biased by the lack of a standardized management protocol, for example, the use of proton pump inhibitor and blood transfusion. Third, endoscopy was performed according to clinical judgment, thus not every dengue-affect patients received endoscopic examination. Fourth, the small number of end stage renal disease cases made the statistical power quite small.

Our study emphasizes that, in addition to older age and thrombocytopenia (platelet count <50 × 10^9^ cells/L), end stage renal disease and previous stroke, with additional comorbidities, are important clinical predictor of GI bleeding in adult patients with DENV infection. More studies, particularly prospective studies are required to validate these findings for better generalization of their clinical utility. Nevertheless, this is the first study to find an association between GI bleeding in dengue and end stage renal disease and previous stroke, with additional comorbidities. Our findings may have impact on screening and identify those with higher risk of GI bleeding, and to keep them for observation and monitoring in hospital. Given the high fatality rate of dengue-affected patients with GI bleeding, early effective intervention in such cases will avoid the otherwise preventable mortality and morbidity. Our study also highlights the need for further clinical studies to develop new protocols for management of dengue in elderly and those with end stage renal disease and previous stroke.

## Supporting information

S1 Table Clinical data of 24 patients with severe gastrointestinal bleeding. (DOCX)
