# Supplementary material for: Characteristics and predictors for gastrointestinal hemorrhage among adult patients with dengue virus infection: Emphasizing the impact of existing comorbid disease(s)
Source: PLoS One. 2018 Feb 20;13(2):e0192919. doi: 10.1371/journal.pone.0192919 (PMC5819790; doi:10.1371/journal.pone.0192919)
Supplement: S1 Table — (DOCX) [file pone.0192919.s001.docx]

**S1 Table. Clinical data of 24 patients with severe gastrointestinal bleeding**

| **Variable** | **Severe GI bleeding (n=24)** |
| --- | --- |
| Mean age (±SD), years | 63.7 ± 9.95 |
| ≥ 60 years | 18 (75) |
| Female | 12 (50) |
| Comorbid condition^a^ |  |
| Type 2 diabetes mellitus | 6 (25) |
| Essential hypertension | 11 (45.8) |
| Non-dialysis chronic kidney disease | 6 (25) |
| End stage renal disease (hemodialysis) | 2 (8.3) |
| Previous stroke | 4 (16.7) |
| Ischemic heart disease | 4 (16.7) |
| Dengue hemorrhagic fever | 24 (100) |
| Grades 1 and 2 | 6 (25) |
| Grades 3 and 4 | 18 (75) |
| Complications |  |
| Pleural effusion, n/total n (%) | 10/19 (52.6) |
| Ascites, n/total n (%) | 5/13 (38.4) |
| Fatal | 7 (29.2) |

Data are presented as numbers (percentages) unless otherwise indicated. GI = gastrointestinal bleeding; n/total n = number of patients/total number of patients with data available.

^a^An individual patient might have more than one underlying disease/condition.
